# Construction of medical equipment-based doctor health monitoring system

**DOI:** 10.1007/s10916-019-1255-z

**Published:** 2019-04-10

**Authors:** Shaogang Wang, Shuai Cheng, Xianghua Zhou, Yeyun Mao, Ying Li, Gang Long, Cong Li, Wei Liu, Xueping Long

**Affiliations:** 1 Department of Urology, Tongji Hospital, Tongji Medical College, Huazhong University of Science and Technology, Wuhan, 430030 China; 2 YouCare Technology Co., Ltd., Wuhan, 430223 China; 3 Department of Physics and Astronomy, University of California, Los Angeles, CA 90095 USA

**Keywords:** Doctor health, Doctor health monitoring system, Physical health indicators, Convolutional neural network, Health status

## Abstract

The health status of doctors has been overlooked by the society and even the doctors themselves, especially those doctors who work long hours. Their attention is always on patients, so they are more likely to ignore their own health problems. Therefore, in this paper, we propose a medical equipment-based doctor health monitoring system (hereinafter referred to as Doc-care). Doc-care can be used as a private health manager for doctors, and doctors can monitor their health indicators in real time while using medical equipment to aid diagnosis and treatment. When the doctor’s health status is neglected, Doc-care can protect the doctor’s health; combining with the convolutional neural network method to detect and grade the doctor’s health indicators, to assess the doctor’s real-time health status. After referring to the doctor’s past health data in the cloud server, giving appropriate advice and predictions about the doctor’s health status.

## Introduction

In October 2017, the Hippocratic Oath was made for the 8th revision and subsequently published in the Journal of the American Medical Association (JAMA), and nearly 150,000 people read the article online (until February 2019). Compared with the previous Hippocratic Oath, it has conspicuously added a “I will value my health, life and ability to provide the highest level of medical care” [1], which reflects the doctor’s emphasis on self-health, only healthy doctors can provide high-quality medical services. In fact, now due to the doctors often overwork and involve with high stresses, irregular diet and work schedule, lack of sufficient time to exercise and rest, thereby leading to the ignorance of their own health; they deal with patients almost every day, and even conduct the clinical treatments to patients. Each clinical diagnosis and treatment is a rigorous process of thinking activities for them, which can be easily affected by the emotional and psychological conditions. Even if there is a slight mistake, serious consequences such as missed diagnosis, misdiagnosis, and surgical operation errors may occur, thereby affecting the quality of medical services and patient treatment effect. In view of this, the health problems of doctors should attract enough attention from society and themselves.

In recent years, the use of digital data such as behaviors and physiology collected in daily life by applying the wearable telemedicine technologies to raise awareness of various physical and mental health outcomes, has received increasing attentions. As early as 2004, Anliker Urs et al. designed a portable telemedicine monitoring device called “AMON” [2], which can continuously collect and evaluate multiple vital signs, intelligently detect medical emergency multi-parameters and wirelessly connect medical care, and provide a wearable care and alarm system for high-risk heart and respiratory patients. Subsequently, Shahriyar and other scholars proposed an intelligent mobile health monitoring system (IMHMS) [3], which provides medical feedback to patients via mobile devices based on biomedical and environmental data collected by sensors. Later, Joao Martinho et al. designed a remotely operated physiological monitoring devicethat completed the measurement and acquisition of three physiological indicators [4]: electrocardiogram, blood oxygen and blood pressure, and sent the waveform to the remote back server via Wi-Fi Internet, the device can be remotely controlled without patient intervention. In recent years, many companies and scholars have focused their research on the implementation and application of cloud computing and artificial intelligence technology in remote health monitoring service systems [5–10], and also achieved certain theoretical and applied results. But the studies on the health of doctors and the study of doctors’ health monitoring systems have not yet begun.

Based on the study of the predecessors’ literatures, the bio-sensor technology was used to collect the doctor’s health index in real time, the cloud computing and artificial intelligence technology were introduced, and the Doc-care system was constructed and verified by experiments. The results showed that the doctor’s physiological indicators obtained in real time in Doc-care were accurate and effective.

## Methods

**Fig. 1:**
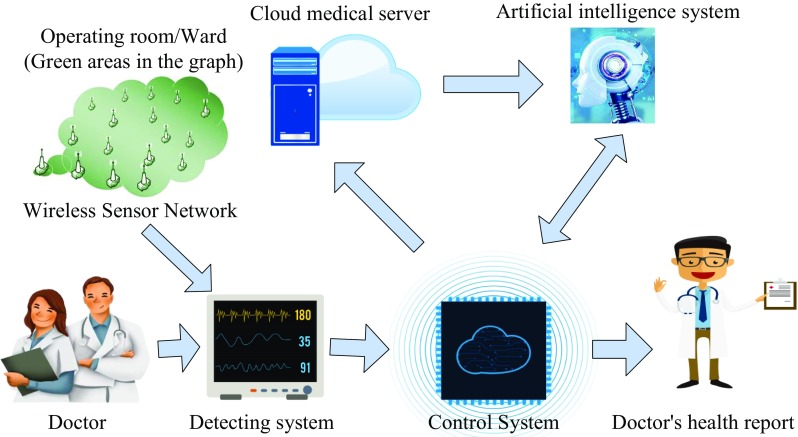
Overall frame structure

Overall framework of Doc-care. Doc-care is based on medical equipment, and this system includes medical equipment, detection systems, control systems, an artificial intelligence system and cloud medical servers [11], as shown in Fig. 1.

As a carrier of Doc-care, medical devices integrated with this system can monitor the health indicators of doctors in real time when they are using the equipment. The detection system is used to collect health indicators of doctors and send the collected data to the control system; the control system and the artificial intelligence system are integrated in the medical equipment. Therein the control system processes the received data, monitors health indicators of doctors in real time and outputs for display, and transmits the data through the communication module to the cloud medical server for storage. The artificial intelligence system is used to analyze a large amount of historical data in the cloud medical server to obtain health indicators of doctors, so as to better assist doctors in understanding their physical condition.

**Fig. 2:**
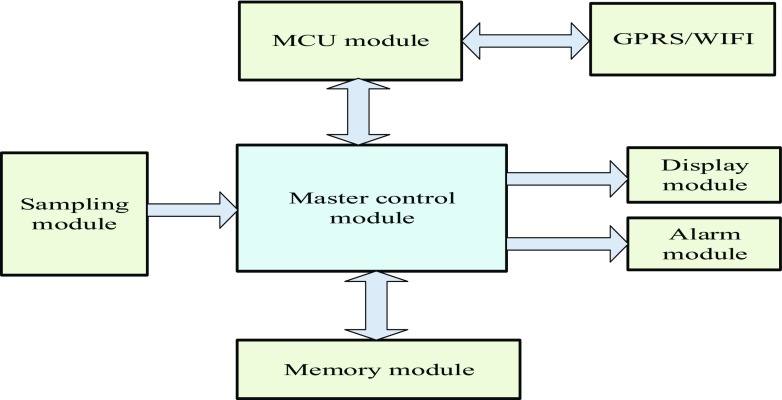
Control system structure

**Doc-care’s control system structure**. The control system consists of the master control module, MCU module, memory module, sampling module, GPRS module, display module and alarm module, as shown in Fig. 2:

Doc-care mainly receives data through the sampling module, transmits the data to the memory module for caching and reads and processes the data through the master control module. The master control module monitors whether the health indicators exceed the alarm limits in real time and outputs for display. If the health indicators exceed the normal alarm limits, the alarm module will produce a warning immediately [12]; in addition, the master control module establishes communication with the.

GPRS through the MCU module and transmits the collected data to the cloud medical server for storage [13].

**Fig. 3:**
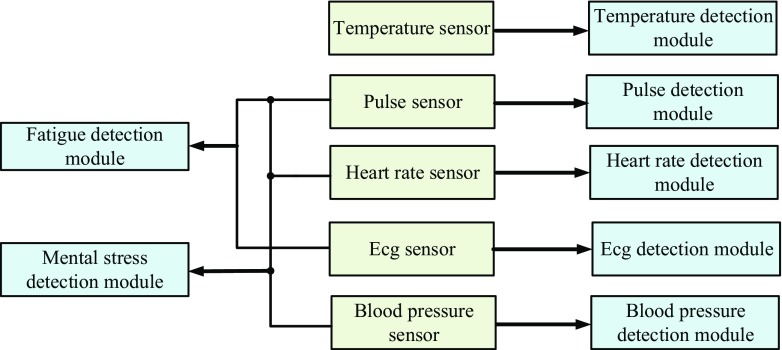
Monitoring system structure

Doc-care’s detection system structure. The detection system mainly detects the physiological signals of doctors, as shown in Fig. 3:

The master control system is used for post-stage function processing, including a pulse detection module, heart rate detection module, temperature detection module, ECG detection module, blood pressure detection module, fatigue detection module and mental stress detection module. As is known, sensor detection module is inevitably affected by various types of noise interference in the process of diagnosis and treatment, so it is necessary to filter and eliminate interference in the process of data collection. Although the hardware filtering technology is relatively mature, it has certain limitations, and all parameters must be set in advance. In recent years, software filtering technology has been widely used. Compared with hardware filtering technology, it is more flexible, simple, efficient and accurate [14].

The pulse detection module collects a doctor’s pulse signal using the pulse sensor, converts it into pulse wave and then into an electrical signal by a signal conditioning circuit for measurement and analysis. The pulse wave can be detected by a doctor’s surface arteries, such as the carotid artery, brachial artery or radial artery [14].

The heart rate detection module converts the original heart rate signal into micro-voltage signal using the heart rate sensor and outputs a square wave with a certain voltage amplitude. The output signal is processed by the signal conditioning circuit into a digital signal to be transmitted to the master control system [15].

The body temperature detection module collects doctor’s body temperature value using the temperature sensor. The body temperature collection mode and technology are very mature and the precision is also very high. There are body temperature measuring sensors such as traditional contacting and infrared inductive measurements, which meet the requirements of simple and rapid measurement [16].

The ECG detection module collects the doctor’s ECG signal using the ECG sensor, and then amplifies, filters, and shapes output using the conditioning circuit. As a manifestation of cardiac activity in the human body surface, the ECG signal is a weak low frequency AC biological signal of the millivolt level, and it includes P wave, R wave, QRS wave and T waves. The corresponding changes of the ECG signal is an important basis for testing doctors’ health indicators [17]. In fact, ECG is used as band image recognition processing, in order to improve the accuracy of band image capture and acquisition, the relevant image algorithm processing is required to ensure the accuracy and efficiency of ECG [18, 19].

The blood pressure detection module collects and quantizes the blood pressure signal using the blood pressure sensor, converts it into a weak electric signal, performs morphological filtering using a low-pass and high-pass filter, and then performs A/D conversion and outputs to the master control system. Blood pressure is one of the most important physiological parameters in doctors’ health indicators, and accurate measurement helps early detection and identification of types of hypertension and better detects doctors’ blood pressure changes at work in real time [20, 21].

The fatigue detection module obtains a fatigue detection result by calculating and analyzing heart rate variability (HRV), pulse rate variability (PRV) and ECG curve using upper-level computer software. Fatigue is classified as mild, moderate and severe fatigue by severity. Once Doc-care detects severe fatigue in a doctor, the alarm system will give a warning message to the doctor [22].

The generation of mental stress is related to factor such as work intensity, environment, mood, and health level, and these factors can be summarized in both subjective and objective aspects. Mental stress tests can start from a medical point of view, and the change in a doctor’s mental stress will objectively affect blood pressure, heart rate, ECG, pulse and other physiological indexes. The upper-level computer software calculates and analyzes the severity of mental stress using the collected data of the doctor’s physiological indexes, and the severity is rated as Grade 1, 2, 3 and 4 [23]; if it is detected that the doctor’s mental stress is above Grade 3, Doc-care will immediately send instructions to the alarm system.

With the high-speed development of the wireless network, doctor’s physiological data from the sensors in the Doc-care system can be combined the technology of wireless sensor network (WSN). Furthermore, through wireless communication mode to form the multi-hops routing and self-organizing network system and each node of the sensor is connected to the wireless network to achieve the efficient detection of the relative information with the support of the network carrier and can be conducted to ensure the effect of the medical work.

WSN has many applications in medical system and health care. Doc-care system combined with wireless sensor network technology can improve the flexibility and timeliness of the system and more efficient diagnosis and treatment assistant for doctors; In the process of surgery, the sensor network nodes of doctors monitor such as pulse, heart rate, blood pressure and body temperature etc., which can timely learn the physical conditions and activities of doctors. When abnormal conditions are found, they can give warnings at the fastest speed to ensure the health and safety of doctors and patients [24–26].

**Fig. 4:**
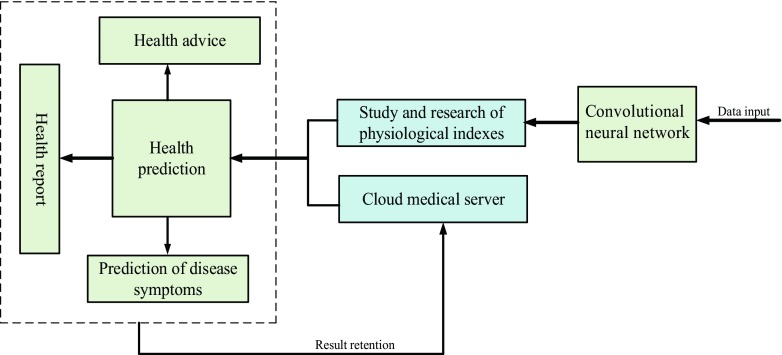
AI system structure

**Doc-care’s artificial intelligence system structure.** The artificial intelligence system of Doc-care comprehensively predicts health indicators, as shown in Fig. 4:

The artificial intelligence system obtains a doctor’s accurate health indicators through data flow [27, 28] input to the cloud medical server. Through accurate consideration and analysis such as deep learning and neural networks, it thereby assists doctors in understanding their health statuses and provides a detailed private health report to each doctor.

Doc-care health indicator identification. The artificial intelligence system in Doc-care reads the data of a doctor’s health indicators stored in the cloud medical server and then predicts and manages the doctor’s health status through deep learning and research in the neural network [29, 30]. Artificial neural networks can more accurately simulate the distribution of multiple types of data in the doctor’s historical records [31, 32]. The structure of convolutional neural network used in this paper includes: an input layer, two-layer cascade convolutional layer, pooling layer, discarded layer and fully connected layer, and then they are classified by Softmax [33]. The input layer converts the input of the physiological indicator data into a two-dimensional matrix form for convolution processing. The convolutional layer is used to extract features from the input matrix. The pooling layer is used to extract the main features. The discard layer is used to receive the parameter of the previous layer, and a part of the parameters are randomly discarded in proportion, which can effectively avoid over-fitting of the model and reduce the model parameters, thereby simplifying the model. The fully connected layer connects all the features and passes them to the Softmax classifier to calculate the correlation between the input feature vector and various physiological indexes, and finally obtains the predicted value of each index. The input layer is the health indicator data of each healthcare provider. We first connect the data of health indicators monitored each time to form a complete data link. We use the matrix T∈ *R*^*S* × *D*^ to display, as shown in eq. (1), where line S represents the data of health indicators for the Sth monitoring, and each healthcare provider is represented as a D-dimensional matrix,

1 $$ \mathrm{T}=\left[\begin{array}{l}D{\mathrm{ata}}_1\\ {}D{\mathrm{ata}}_2\\ {}\kern0.48em \vdots \\ {}D{\mathrm{ata}}_S\end{array}\right]=\left[\begin{array}{l}{x}_{1,1}\;{x}_{1,2}\cdots {x}_{1,D}\\ {}{x}_{2,1}\;{x}_{2,2}\cdots {x}_{2,D}\\ {}\vdots \kern0.72em \vdots \kern0.36em \ddots \kern0.36em \vdots \\ {}{x}_{S,1}\;{x}_{S,2}\cdots {x}_{S,D}\;\end{array}\right]\kern0.24em $$

The convolutional layer contains convolution kernels of multiple sizes, but the depth of each convolution kernel is equal to the depth of the input matrix. If the depth of the kth convolution kernel is C, the convolution kernel is expressed as *B*^*k*^∈*R*^*C* × *D*^, as shown in eq. (2). In this paper, a two-layer cascade convolutional layer structure with the convolution kernel size of 3 × 3 is used, which can achieve a larger receptive domain with fewer training parameters.

2 $$ {B}^k=\left[\begin{array}{l}{b}_{1,1}^k\;{b}_{1,2}^k\cdots {b}_{1,D}^k\\ {}{b}_{2,1}^k\;{b}_{2,2}^k\cdots {b}_{2,D}^k\\ {}\vdots \kern0.72em \vdots \kern0.36em \ddots \kern0.36em \vdots \\ {}{b}_{C,1}^k\;{b}_{C,2}^k\cdots {b}_{C,D}^k\;\end{array}\right]\kern0.24em $$

Convolution operations represent the process of feature extraction from the input matrix. When ${b}_{1,1}^k$ convolves with ${x}_{1,1}$, the convolution kernel extracts feature *T*_1 : *C*_ from ${\mathrm{e}}_1^k$, as shown in eq. (3),

3 $$ {e}_1^k=f\left\{\sum \limits_{i=1}^C\sum \limits_{j=1}^D{b}_{i,j}^k\cdot {x}_{i,j}+{\varepsilon}_{i,j}^k\right\}, $$

Wherein, ${b}_{i,j}^k$ represents the vector in Row i and Column j, ${\varepsilon}_{i,j}^k$ represents the bias term, f is a nonlinear function, and the relu activation function is used as the nonlinear function in this paper, as shown in eq. (4):

4 $$ f(x)= relu(x)=\max \left(0,x\right) $$

The convolution process is the product of two frequency domain functions, and the convolution kernel *B*^*k*^ slides from top to bottom in a certain step size *T*_*e*_ to calculate the eigenvector of each part. Finally, the convolution kernel *B*^*k*^ extracts the eigenvector *E*^*k*^, as shown in eq. (5):

5 $$ {E}^k={\left[{e}_1^k,{e}_1^k,\cdots, {e}_{\frac{S-C+1}{T_e}}^k\right]}^T $$

The pooling layer compresses the input feature map. This makes the feature map smaller to simplify the network computing and, on the other side, feature compression is performed to extract the main features. The max pooling operation is used on feature *E*^*k*^ to find the maximum value. Assuming that the height of the pool kernel is *C*_*p*_, the output is eq. (6). When eqs. (7) and (8) are satisfied simultaneously,

6 $$ {M}^k={\left[{m}_1^k,{m}_1^k,\cdots, {m}_{S_p}^k\right]}^T. $$

Thereinto,

7 $$ {m}_i^k=\max \left({e}_i^k,{e}_{i+1}^k,\cdots, {e}_{i+{C}_p-1}^k\right). $$

8 $$ {S}_P=\frac{\frac{S-C+1}{T_{\mathrm{e}}}-{C}_p+1}{T_P} $$

After all the pooling is completed, the complete eigenvector RT is obtained by joining the first and last eigenvectors of convolution pools in each layer, as shown in the equation (9):

9 $$ {R}^T=\left[{r}_1^T,{r}_2^T,\cdots, {r}_L^T\right]. $$

Thereinto, *r*_*k*_ = *M*^*k*^ and *L* represents the number of features.

All the extracted features *R*^*k*^ are retained for a part of parameters according to the proportion of *p*, as shown in the formula (10) *Bernoulli* represents Bernoulli distribution, *Bnl* is the vector of element 0 or 1, the proportion of 1 accounted is *p*, the length of vector is equal to the length of *R*^*k*^, when *Bnli* is 0 in the training process, the corresponding neurons are invalidated.

10 $$ Bnl\sim Bernoulli(p) $$

The total eigenvector of the health indicator data is obtained, as shown in eq. (11):

11 $$ {Q}^T=\left[{q}_1^T,{q}_2^T,\cdots, {q}_{S_{p.L}}^T\right]. $$

At *q*_*i*_ = *Bnl*_*i*_ · *R*_*i*_ the same time, *L* represents the number of features.

The fully connected layer connects all the features, and by defining the weighting matrix *W*, calculates the weighted sum of each feature element, thus obtaining the final feature representation of the Sth input data of health indicators, as shown in eq. (12):

12 $$ y=W\cdot Q+{\varepsilon}_f. $$

Finally, the output y is sent to the Softmax classifier to obtain a predicted value for each health indicator [34].

## Results

Monitoring health indicator data. The detection system detects the doctors’ physiological signals. In Table 1, the pulse/heart rate and body temperature are detected three times to obtain the average final values, which improves the measurement accuracy. For blood pressure, the actual data is measured by the blood pressure sensor, and the results of low, normal and high pressure are given by analysis using the upper-level computer software. The fatigue detection results are obtained by calculating and analyzing heart rate variability (HRV), pulse rate variability (PRV) and ECG curve using upper-level computer software. Fatigue is classified as mild, moderate and severe by severity. For mental stress, the upper computer software calculates and analyzes the severity of mental stress using the collected data of the doctor’s physiological indicators, and the severity is rated at Grade 1, 2, 3 and 4. Finally, the data is trained and tested by the convolutional neural network method. The position distribution of the doctor’s physical health indicator in the feature space is analyzed, and the possibility of the physical condition of the doctor can be calculated. The prediction results are shown in Table 1.

**Table 1.** Health index test data.

	Pulse/heart rate (bpm)			animal heat (°C)			BP (mmHg)		fatigue	ECG	mental stress
SN	1	2	3	1	2	3	SBP	DBP	1	1	1
index	78	66	82	36.5	36.4	36.6	102	86	Mild	Normal	Four
result	75			36.5			Normal		Abnormal	Normal	Normal
advise	Have mild fatigue, pay attention to rest										

## Discussion

The automatic extraction of physical health indicators is of great significance and value to clinicians and their diagnosis of patients. The Doc-care proposed in this paper can be considered a private health manager for doctors. It can completely record all the doctor’s health indicators and form a health report. It can also provide appropriate guidance and suggestions. The most representative function of Doc-care is to monitor the health indicators of doctors in real time when they are using medical equipment to assist in diagnosis and treatment. The complete doctor health monitor data is recorded in Table 1. The feedback of all physiological indicators are provided to the doctor in real time, and the corresponding monitoring results (normal or abnormal) are provided. The pulse/heart rate and temperature data are collected three times to obtain average values to ensure the accuracy of monitoring data. Systolic blood pressure of 102 mmHg and diastolic blood pressure of 86 mmHg are in the normal ranges of blood pressure. The severity of fatigue is moderate, calculated based on heart rate variability (HRV), pulse rate variability (PRV) and ECG curve, and when Doc-care detects an abnormal severity of fatigue, it will alert the doctor to rest and ensure a good mental state while working. Diagnosis and treatment by a doctor in good physical condition is a better safeguard for patients. Table 1 accurately shows the health indicators to the doctor. Each group of data monitoring results can promptly report the doctor’s health status, demonstrating the effective and feasible construction of Doc-care. However, research and study of Doc-care is a difficult and long-term task. In the medical industry, people always focus on patients and overlook the health problems of doctors, but Doc-care is just the opposite. This paper proposes the construction of Doc-care, which will open the door to medical equipment with doctor health monitoring at its core. This will have profound influence on future medical device development. In the future, more user-friendly and more advanced technologies will be applied towards caring for the health of doctors.

Although we have achieved the expected results, there are still some limitations to be considered in the current exploratory study. Firstly, in this paper, the introduction of wireless sensor network system can improve the detection efficiency. In addition, the better all-round detection of doctors’ health status is of great significance in the development of medical equipment industry. Secondly, this article used only a few of the most common physical health indicators and did not fully reflect the health status of the doctor. Therefore, in the future studies, we will try more studies, including more types of physical health indicators, as well as human-related excreta parameters. Lastly, doctors’ health status with different ages, genders, departments, clinical working hours, and geographical distribution will also be included in the next study works of our team.
